# Internet of Things Task Migration Algorithm under Edge Computing in the Design of English Translation Theory and Teaching Practice Courses

**DOI:** 10.1155/2022/9538917

**Published:** 2022-06-16

**Authors:** Shangying Guo

**Affiliations:** ^1^School of English, Xi'an Fanyi University, Xi'an City 710105, China; ^2^University of Perpetual Help System DALTA, Alabang-Zapote Avenue, Pamplona 3, Las Piñas City, Mero Manila 1740, Philippines

## Abstract

This study aims to improve the quality of English teaching in contemporary colleges and universities, so as to cultivate more English translation talents. Taking corpus, English translation, and teaching practice as the main research horizons, this study analyzes the application of master of translation and interpreting (MTI) course design, English translation, and task migration by combining the task migration algorithm under Internet of Things (IoT) with the self-built corpus, so as to realize the cultivation of translation talents and design of teaching practice. A translation corpus is constructed, a two-way interactive online course is designed, and the experimental results of the complete local migration algorithm (CLM algorithm), random migration algorithm (RM algorithm), and greedy heuristic migration algorithm (GHM algorithm) used in English teaching practice courses are analyzed and compared. The experimental simulation results reveal that the GHM algorithm proposed in this study shows good system stability, its duration is 60% better than that of the CLM algorithm, and its system throughput is increased by 50% compared with the CLM algorithm. In addition, the maximum delay time has little effect on the system throughput. When the system time slot length is fixed at 20 ms, the user migration rate of the genetic algorithm is the highest under the different total numbers of users. In addition, in view of the wide application of neural network in English translation teaching, this study establishes an English translation evaluation model based on the combination of particle swarm optimization (PSO) algorithm and neural network and compares it with the traditional neural network model in simulation experiments. The results show that the addition of PSO algorithm can effectively improve the convergence speed of artificial neural network (ANN), reduce the training time of the model, and improve the accuracy of the ANN network. Using the PSO algorithm to train the neural network, the optimal solutions of different particle swarms can be obtained, and the error is small. The PSO-ANNs model can promote the quality of English translation teaching and improve the English translation ability of the students. Therefore, applying the task transfer algorithm and PSO algorithm to the practice of English translation teaching has greatly improved the efficiency of the English classroom. To sum up, this study provides new ideas for the curriculum design of contemporary college English teaching and has reference value for the cultivation of college English translation talents.

## 1. Introduction

With the development of today's economic globalization, the professionalization of the ancient profession of translation is getting higher and higher. Translation services belonging to the language service industry, such as professional document translation, interpretation and conference translation, editing and typesetting, multimedia translation and production, website and software localization, and language service outsourcing, have gradually entered the business scope of translation companies. The standardization and institutionalization of translation project management, translation customer service, and translation quality monitoring are also getting higher and higher, with obvious service industry characteristics and increasingly clear professional requirements. This requires employees in this industry to have special professional qualities, abide by professional ethics, understand professional characteristics, and possess professional abilities and qualifications. Of course, they also need industry associations and other organizations to formulate industry standards and practice rules, establish access mechanisms, and carry out training. The most important teaching goal for English teaching in contemporary colleges and universities is improving the translation ability and translation cognitive ability of students [1]. Translation-related teaching theories can not only improve the quality of English teaching but also lay a reliable foundation for the overall development of quality education, which is the essential way to quality education [2]. Therefore, a complete translation research theory has been formed in the current teaching system, bringing together various translation skills and strategies required in the translation process, which is the theory summed up by many scholars after countless practice. It can not only help students find the laws in translation learning and solve high-frequency problems in translation but also provide efficient guidance for translation practice of students [3]. The student who wants to upgrade his translation ability has to start from the theoretical basis of translation. For teachers, English translation can help them arouse the learning interest of students and guide them to learn more deeply [4]. In English teaching, the proficiency of using translation theory represents the English translation ability of students or teachers to a certain degree [5].

The choice of translation theory teaching content is based on the teaching objectives of English translation theory. Only clear teaching objectives can be integrated into the classroom in actual teaching work [6]. The main goal of college translation theory in English teaching is enlightening the translation learning of students, so that they can learn subjectively and independently, and then applying the learned translation theory knowledge in actual life and work, forming a virtuous circle of improving their translation ability [7]. In English class, teachers still tend to adopt the traditional teaching plan, which requires assigning the translation task to the students, and wait until the students complete the task before commenting on it in the classroom [8]. Generally, there is a long-time interval between the completion of the homework by the students and the explanation by the teacher. Thus, the students will not get timely feedback information, the motivation of learning will decrease, and the quality of learning will only decrease. In addition, this traditional teaching mode and classroom interaction methods are very single, and the classroom atmosphere is boring and depressing, which often reduces students' interest in learning to a large extent [9]. Such problem can be effectively solved through online classes. The resources of the cloud data center are distributed near the mobile terminal, and the multiaccess edge computing (MEC) platform is built to manage the computing and storage resources together. On the contrary, mobile terminal equipment migrates computing, storage, and other functions to the MEC platform to solve insufficient computing power, which is the principle of edge computing [10]. English translation teaching theory and edge computing are combined, and the mobile edge technology is applied to existing English teaching, providing valuable research ideas for current English teaching [11]. In recent years, the English translation model based on artificial intelligence (AI) has been a hot research topic. The researchers established a learning ability analysis model by collecting a large amount of learning data and used the model to analyze the characteristics of English translation. At present, edge computing algorithms and neural network algorithms are widely used in teaching. Wu and Song (2020) [12] analyzed the challenges of video distribution based on edge computing from three aspects of edge computing, storage, and network resources for the distribution of teaching surveillance videos; then, a teaching surveillance video content distribution framework based on the storage and computing power of network edge computing is proposed. Sun (2020) [13] proposed a system resource allocation method based on power iteration, which takes the throughput of the offloading process as the objective function and achieves the optimal allocation of normal power through iterative optimization. The results show that the method is effective and useful. Bao and Yu (2021) [14] proposed an online and offline hybrid teaching quality evaluation method based on mobile edge computing to strengthen the online and offline hybrid teaching of sports. Therefore, one of the innovations of this study is to use the IoT task migration algorithm under edge computing to study English translation theory and design teaching practice courses. They designed a two-way interactive online course and provided a comprehensive review of complete local migration (CLM), random migration (RM), and greedy heuristic migration (GHM) in English teaching practice. The experimental results were analyzed and compared. In addition, with the continuous development of algorithms and artificial intelligence, various algorithms are also applied to teaching. Sun and Wang (2020) [15] applied the PSO algorithm and neural network to English teaching, and the results showed that this model can improve the quality of English translation teaching, making teaching and learning a win-win situation. Olguín et al. (2018) [16] proposed a new method for measuring the efficiency of machine translation based on evolutionary algorithms and used the translation accuracy of English text sets to Spanish texts as a simulation benchmark and explored the reverse process. The results show that the PSO algorithm can be used for the translation of multilingual sentences with only one identifier. Another innovation of this study is to establish an English translation evaluation model based on PSO algorithm and neural network and select real teaching samples to test the model to verify the feasibility of the model.

It is known from the current research that most of the studies of English corpus development and the master of translation and interpretation (MTI) discipline focus on one aspect rather than combining them. Therefore, the work of this study is mainly divided into two parts. The first part is the combination of task transfer algorithm under the IoT and self-built corpus. The application of curriculum design, English translation, and task transfer for the master of translation and interpretation (MTI) is analyzed, so as to realize the cultivation of translation talents and the design of teaching practice. Then, a translation corpus is constructed, and a two-way interactive online course is designed. In the second part, the PSO algorithm is combined with ANNs to establish an English translation evaluation model based on PSO-ANNs.

The contribution of this study is reflected in the research of English translation teaching practice, corpus construction method, and task transfer algorithm technology. It constructs an English-Chinese/Chinese-English bilingual translation corpus and designs a two-way interactive online English translation course combined with the systematic task transfer algorithm in the MEC scenario. Compared with the completely local transfer algorithm and the random transfer algorithm, the greedy heuristic transfer algorithm proposed in this study has higher performance in English teaching practice courses. The innovation of this study can be summarized as two points. The first point is to use the IoT task migration algorithm under edge computing to study English translation theory and design teaching practice courses. The second point is to establish an English translation evaluation model based on the PSO algorithm and neural network.

## 2. Research Theoretical Analysis

### 2.1. English Translation and Teaching Practice

In recent years, the increasing frequency of international exchanges and the continuous improvement of tourism policies have promoted the sustainable and rapid development of China's tourism industry. The application of technologies such as big data, cloud computing, and the IoT has also spawned smart tourism. With the rapid progression of international tourism, the translation and translation talents have gradually become imperative [17]. The information delivery with appropriate time, appropriate content, and appropriate channels is critical to enhancing the experience of inbound international friends [18]. The corpus is one of the powerful platforms which provides effective information. The combination of corpus and translation is of great help to the cultivation of MTI translation talents and the design of teaching practice [19]. According to the language type of the collected data, the corpus is classified into a monolingual corpus and a bilingual or multilingual corpus. The former uses only one corpus and collects many language examples of native speakers, while the latter is a corpus composed of two or more language texts, with corresponding or parallel forms, analogous forms, and translation forms [20].

As an analog corpus, the monolingual corpus is often ignored in the parallel text research [21]. The construction of a Chinese-English monolingual corpus can support MTI students' simulated text and data research translation texts as well as authoritative reference source and target languages in terms of talent training. A high-quality monolingual corpus provides a large number of illustrations for MTI classroom teaching in teaching practice, which is used as a text supplement.

### 2.2. Task Migration in Edge Collaboration

**Figure 1:**
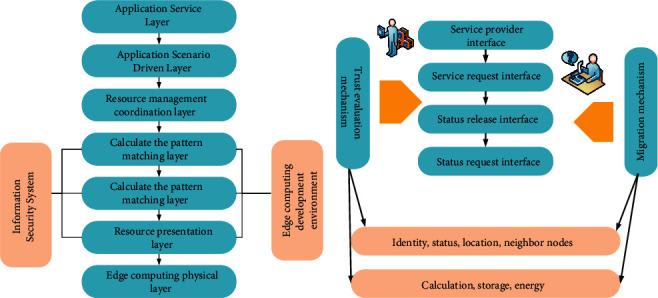
The system structure and units of edge computing: (a) system structure of edge computing; (b) units of edge computing.

The edge computing unit describes the functions and characteristics of the terminal node in the MEC system after resources encapsulation [22], which includes the external interaction part and the internal application part [23]. The external interaction part covers the service provision and request interface, the resource request, and release interface, and the internal application covers its own status, identity, location information, and neighbor nodes. In addition, some edge computing units with powerful hardware performance also show migration mechanisms and node trust evaluation mechanisms [24]. The system structure and units of edge computing are shown in Figure 1.

Edge computing allows end devices to migrate storage and computing tasks to network edge nodes, such as base stations, wireless access points, and edge servers. While meeting the computing capability expansion requirement of the terminal device, it can effectively save the transmission link resources of the computing task between the cloud server and the terminal device. Edge computing is mainly composed of four layers of functional structure: core infrastructure, edge computing center, edge network, and edge devices.

The edge server carries most of the functions of the cloud computing center in the cloud computing mode and can provide services and computing support to each edge terminal, which can lay a solid foundation for the progress of mobile computing [25]. However, the network edge is affected by factors such as environment and hardware, and there is no way to increase the settings of the edge server [26]. As a result, various terminals at the edge of the network begin to perform collaborative tasks. The advantages of such processing include increasing the processing speed of tasks, balancing terminal load and energy consumption, and increasing the utilization rate of system resources. However, challenges and opportunities coexist. There are many factors in designing multiedge terminal tasks, so it is very difficult to design a coordination mechanism for multiedge terminal tasks [27].

**Figure 2:**
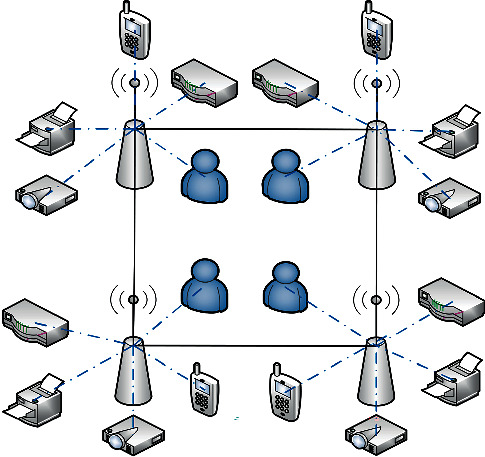
Diagram of the system link model.

In the scenario of multiuser collaboration, the edge computing task offloading network connection model is shown in Figure 2.

The characteristics of the link system are summarized as follows. ① The connection between the incoming line and the outgoing line must go through one or several interlevel connections, and this connection or connecting device is a link. ② The link and the selected outgoing line are occupied at the same time. ③ When the lines are selected, only the links that can be connected to idle outgoing lines should be selected. This method of line selection is called conditional selection.

A system link model is constructed to improve the overall throughput of the system to reduce the network load of core gateways such as cloud servers in the future big data environment. The working principle of this model is sinking the server to the edge of the network, which greatly shortens the transmission distance compared with gathering all tasks in the cloud center for processing [28]. The node only needs to transmit its own information such as task queue, node resources, and node status to the edge server. At this time, the system uses the corresponding uninstall strategy to decide task migration. This system model can greatly reduce the amount of data transmitted by core gateways such as MEC servers in the scenario of multiterminal edge collaboration, thereby reducing the network load and system delay [29].

### 2.3. Algorithm Design of Task Migration

The weighted Euclidean distance of the calculation cost *T*_*pt*_ and transmission cost *T*_*ts*_ for node *n*_*i*_ to migrate to node *n*_*j*_ and the credibility *t*_*fj*_ of node *n*_*j*_ is calculated in the following equation, which is called the matching degree *D*_*ij*_ between the node task and service node:

(1) $$\begin{array}{c} D_{ij}=\sqrt{W_{1}T_{pt}^{2}+W_{2}T_{ps}^{2}+W_{3}trj^{2}}. \end{array}$$

In equation (1), *W*_*1*_, *W*_*2*_, and *W*_*3*_ are the corresponding matching weights of the calculation cost *T*_*pt*_, transmission cost *T*_*ts*_, and credibility *t*_*fj*_ of node *n*_*j*_ in the actual environment, respectively. According to the actual environment of the system, different values can be assigned [30].

To facilitate the comparison of the performance of the three migration methods of CLM algorithm, RM algorithm, and GHM algorithm, the parameters must be controlled to ensure that irrelevant factors have no influence on the judgment of system performance. The experimental simulation data is shown in Table 1.

In Table 1, *K* refers to the number of IoT terminal nodes, the number of base stations refers to the number of smart base stations, and *P*_*ij*_ represents the probability that node *ni* fails to provide services to node *nj*. *c*_1_ and *c*_2_ refer to the link cost of tasks migrating among nodes under the same base station and that under different base stations, respectively. *S*_*ni*_ refers to the number of tasks that IoT can perform in each time slice, and *a*_*ni*_ means the number of IoT node tasks in each time slice.

### 2.4. PSO Algorithm

**Figure 3:**
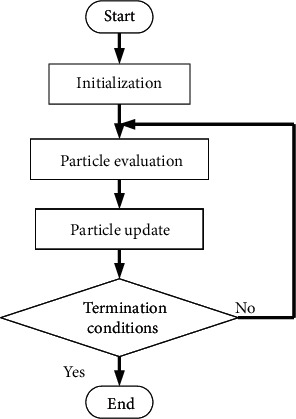
PSO algorithm.

PSO is a swarm optimization algorithm inspired by the foraging behavior of birds. In the population of PSO, individual particles evaluate their own position information in each round of evolutionary iterations by searching for solutions to multidimensional problems in the space. Throughout the group search process, the particles share their “optimal” position information. Then, they use their memory to adjust their own speed and position, constantly comparing and following the candidate problem space solutions. The optimal solution or local optimal solution is found.  Figure 3 shows the PSO algorithm.

The goal of PSO is to make all particles find the optimal solution in a multidimensional volume. All particles in space are first assigned initial random positions and initial random velocities. The position of each particle is then advanced in turn based on its velocity, the known optimal global position in the problem space, and the known optimal position of the particle. As the computation progresses, by exploring and exploiting known vantage points in the search space, particles cluster or aggregate around one or more optimal points. The mystery of the algorithm design is that it retains two pieces of information, the optimal global position, and the known optimal position of the particle. Subsequent experiments reveal that retaining such information has a better effect on faster convergence speed and avoiding prematurely falling into a local optimal solution. This also lays the foundation for the improvement direction of the subsequent particle swarm optimization.

The evolution equation of the basic PSO algorithm is as follows:

(2) $$\begin{array}{c} v_{ij}\left(t+1\right)=\lambda v_{ij}\left(t\right)+c_{1}\cdot r_{1}\cdot \left(b_{ij}\left(t\right)-p_{ij}\left(t\right)\right)+c_{2}\cdot r_{2}\cdot \left(g_{ij}\left(t\right)-p_{ij}\left(t\right)\right),\\ x_{ij}\left(t+1\right)=v_{i_{ij}}\left(t+1\right)+x_{ij}\left(t\right). \end{array}$$

In the above equation, *v*_*ij*_ is the velocity of the particle, *p*_*ij*_ is the position of particle *i* in *j* dimension, and *λ* is the inertia weight.

According to the analysis of the particle search path, to ensure the convergence of the PSO algorithm, the particles continuously approach their local attractors during the particle search process. The equation is expressed as follows:

(3) $$\begin{array}{c} p_{i,j}\left(t+1\right)=p_{ij}\left(t\right)+\beta \cdot v_{ij}\left(t+1\right). \end{array}$$

In the above equation, *t* is the current iteration number. *v*_*ij*_ represents the velocity of particle *i* in *j* dimension. *v*_*ij*_ ∈ [−*v*_max_, *v*_max_], where *v*_max_ is the maximum speed allowed by the particle. *p*_*ij*_ represents the position of particle *i* on *j* dimension, *p*_*ij*_ ∈ [−*p*_max_, *p*_max_], where *p*_max_ is the maximum space position that the particle is allowed to move. *λ* is the inertia weight, used to balance global search and local search. *b*_*ij*_ represents the individual extreme value of particle *i* on *j* dimension. *g*_*ij*_ represents the global extreme value of particle *i* on *j* dimension. *c*1 and *c*2 are acceleration factors that characterize the ability of particles to self-summarize and learn from high-quality particles in the group. *r*1 and *r*2 represent random numbers between [0, 1]. *β* is the constraint factor, which is used to control the weight of speed.

## 3. Application of Task Migration Algorithm in English Teaching

### 3.1. Construction of Translation Corpus

The data collected in this study is used for research, and the translation quality in the parallel corpus must be guaranteed, so the data collector must have a certain degree of bilingual language ability in Chinese and English [31]. The data of this corpus comes from online electronic texts and leaflets produced by authoritative media such as Hujiang English, China Daily, British Broadcasting Corporation (BBC), published books, official websites of tourist attractions, and bilingual subtitles and audio of tourist documentaries [32]. Most of these bilingual corpora are based on Chinese-English translation. At present, about 100,000 English words, 250,000 Chinese words, and 70,000 bilingual words have been collected. Moreover, since research is a process of continuous development, storage capacity will not be static and will continue to expand over time.

During the construction of corpus, the input of a large number of corpora will inevitably lead to phenomena such as misplacement, garbled characters, and symbol errors [33]. At that time, the corpus should be cleaned up. The corpus cleaning is mainly divided into text digitization and text organization. Although the current software applications are quite mature, they still cannot reach 100% accuracy. Therefore, the text after software conversion will inevitably appear garbled and misplaced, and there will be blank lines. Due to the large amount of text, text Processing Master 8.0 is used for text batch processing, which contains functions, such as deleting all blank lines, adding characters in the first line in batches, and handling special characters, which are very helpful in text organization. After the software is operated, the text will be manually checked and processed to examine the text omissions, vocabulary spelling, and other errors that cannot be recognized by the machine to ensure its high cleanliness.

Corpus labeling refers to the division of corpus components for further retrieval and research, which is called part-of-speech labeling. Since the corpus contains bilingual languages, there are differences in labeling methods for different languages. When the English corpus is labelled, the corpus part of speech is divided and abbreviated, and the text is annotated with a tokenizer. However, the machine labeling cannot reach 100% accuracy, so manual verification is required [34].

When the Chinese texts in the corpus are labelled, the parts of speech should be labeled on the ICTCLAS platform firstly. Then, the corresponding rhetorical means are labelled on the corpus according to a prominent rhetorical feature in the tourism discourse. Because there is no ready-made software to complete such marking, it needs to be done manually. During manual review, the rhetorical features contained in the sentence are obtained through the study of the phonetic, vocabulary, and grammatical features of the text. Different rhetorical devices are specifically labelled with sentences as a unit. The corpus mainly adopts autonomous coding methods, such as expressing similes, expressing puns, and expressing parallelism. Although manual coding is a huge project, once these annotations are completed, they will provide evidence for us to reveal the differences in the rhetorical usage of the English and Chinese tourism texts and the differences in the aesthetic concepts behind the texts. This is of great help to comparative rhetoric and comparative aesthetics [35].

Corpus alignment is mainly for parallel corpora of Chinese and English texts, and the main steps are sentence division and sentence coding. The Chinese in the bilingual translation corpus is divided into five wildcards in words, which are ^p, ? ^p, ! ^p, “ ^p”, and : ^p. Replacing the five wildcards with the corresponding five punctuation marks can achieve a better sentence effect. The sentence division of the English corpus only requires four wildcards ( ^p, ? ^p, ! ^p, and “ ^p”). The above steps are repeated again. Finally, the text is imported into Word Smith Tool 6.0. It can view the text that has been divided by clicking the “right button” next to the corpus and then clicking “view.” The text can be saved and exported by clicking “Save.”

### 3.2. Task Migration in MEC Scenario

Multiuser collaborative service is only one of many application scenarios of edge computing. The task migration model of migrating multiuser tasks to the same edge cloud base station is analyzed in this study. Smart classrooms, smart phones, smart glasses, smart bracelets, and other smart IoT terminals have to perform complex and quantitative computing tasks in the development of future application-oriented businesses such as smart classrooms, smart transportation, smart cities, smart homes, and smart tourism. However, the battery energy of these smart IoT devices is often very limited, which cannot meet the life and work needs of human beings well. Edge computing well embodies the characteristics of distributed computing. The calculation is performed in the local area network (LAN) without transmitting the computing data or raw data to the cloud for processing through the network. It can reduce the computing load of the terminal device and can respond to the user request quickly.

**Figure 4:**
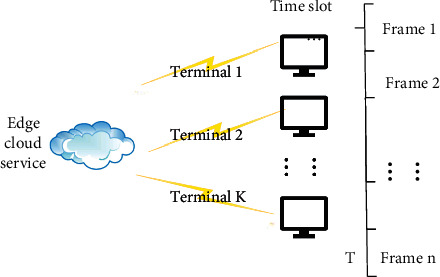
System model in the MEC scenario.

The system model for calculating migration in this study is the time division multiple access MECO system in long-term evolution (LTE). The total migration time slot *T* is divided into disjoint time frames firstly, and then each different frame is divided into different channels. The end users correspond to different channels, and the edge server uses the different channels to distinguish the end users, so as to realize the network link of multiple terminals and a single edge server. The MECO migration system with *K* as the number of end users is shown in Figure 4.

The operation process of the system in Figure 4 is as follows. In Step 1, the end user accepts the task and uploads its own information to the edge server. In Step 2, the edge server makes a decision on task scheduling and sends the decision result. In Step 3, the terminal user receives the decision result and decides whether to migrate its own tasks to the edge server according to the decision result. In Step 4, the edge server receives the tasks of each terminal for response processing and returns the result to the end user. In this scenario, the tasks received by end users in each time slot are regarded as complete and indivisible; that is, tasks can only be computed locally or migrated completely.

**Figure 5:**
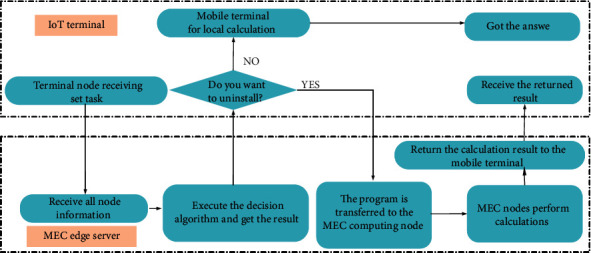
The migration steps of the MEC system.

Computing migration includes five modules of application perception, perception collection, task splitting, overall scheduling, and terminal execution. The computing migration decision process involves the number of end users, edge servers, channels, and bandwidth. Moreover, it may move with the end user's location. Therefore, the bandwidth, channel gain, communication noise, and terminal transmission power of the system are considered jointly in this study to establish a migration model for edge computing in the case of multiple terminals and multiple edge service servers. The migration steps of the MEC system are shown in Figure 5.

A mobile device is composed of a migration decision unit, a local processing unit, and a transmission unit. The mobile device migrates some computing-intensive computing tasks to the MEC server for execution through the transmission unit, so as to solve the limited computing power of the mobile terminal. The MEC server is a virtual machine device deployed at a wireless access point and installed in a small data center, which can provide powerful IT services for mobile devices.

The energy consumption optimizations in multiterminal single edge service computing migration of CLM algorithm, RM algorithm, and generative algorithm are simulated to analyze the energy consumption performance of the three migration algorithms. Massively controlled experiments are performed. The simulation experiment parameters for MEC calculation migration are shown in Table 2.

In Table 2, *K* refers to the total number of IoT terminal users in system. *T* is the total migration time slot. *C*_*k*_ is the calculation consumption of the terminal calculation *k*. *σ* represents the variance of Gaussian white noise. *B* refers to the channel bandwidth. *P*_*k*_ refers to the transmission power of the IoT terminal. *S*_*k*_ is the computing power of the terminal calculation *k*, and *e*_*k*_ is the energy consumption of each cycle for local calculation of terminal *k*.

### 3.3. Task Migration for the Class

The two-way interactive online education model is based on the first generation of online education, which increases the interactive feedback link, considers the learner's learning experience and learning effect, and emphasizes the interactive nature of learning and the use of technology to deliver education and teaching content. It also contains feedback from learners and organizes students to communicate and discuss. This education model mainly focuses on three-screen courses, massive open online courses (MOOCs), and video open courses.

**Figure 6:**
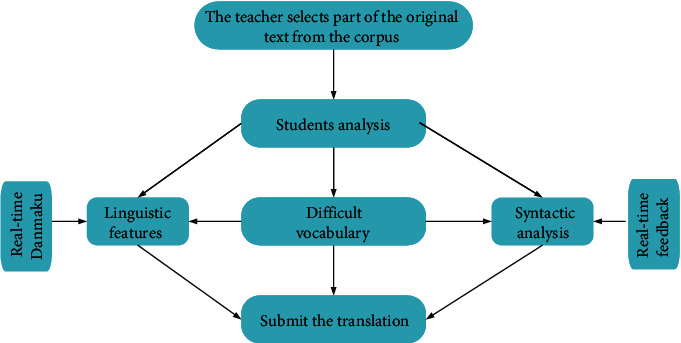
Process for two-way interactive online education course.

In addition to the test questions, assignments, and learning works contained in the traditional two-way interactive education model, the translation course design is supplemented with a new real-time barrage function. According to general functions, MTI students use the tools of the corpus, such as retrieval, clustering, collocation, vocabulary, and keyword list to analyze and translate the original text in the parallel translation corpus. Many comments may suddenly appear on the screen within a certain second and float on the screen like a horizontal version. This kind of online comment method is called “barrage” by netizens. At present, barrage videos are mostly used in media fields such as movies, television, and live broadcast platforms. However, the potential value of barrage videos such as distance education and online learning has received little attention and exploration by domestic and foreign researchers. One of the major disadvantages of traditional two-way interactive online education is the inability to get real-time feedback and interaction from students. The addition of the real-time barrage function can reflect and solve problems in the learning process in a timely manner, greatly improving the learning efficiency and enhancing the participation of MTI students. Compared with other majors, MTI focuses on the active participation of students. Therefore, online teaching based on real-time barrage is very suitable for actual teaching of MTI. The teaching content is detailed and practical, which further promotes the innovation of MTI teaching. The design of the translation course is shown in Figure 6.

The two-way interactive teaching mode in the network environment shown in Figure 6 can fundamentally change the role of teachers (from lecturers to instructors), change the status of students (from receiver to the main body), change the function of the media (from presentation tool to the recognition tool), and change the teaching process from the process of logical analysis and teaching to the process of acquiring knowledge and cultivating abilities by discovering and exploring problems.

**Figure 7:**
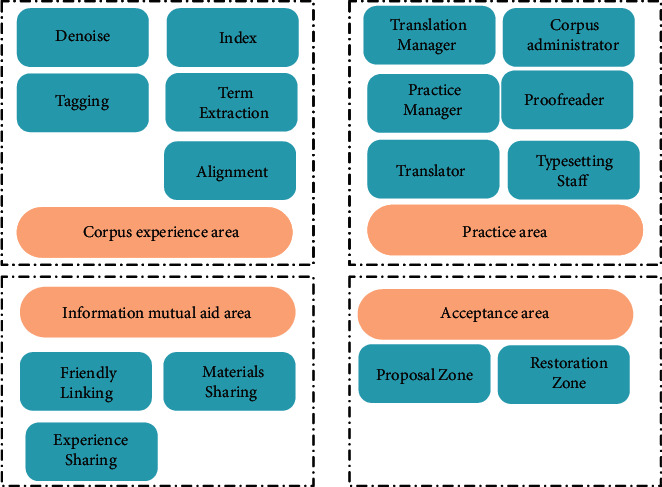
The plan of the translation corpus.

A translation workshop platform suitable for students and teachers is constructed based on three self-built tourism corpora. The users of the platform are classified into teachers, students, and managers. To ensure the security of the platform, teachers and students have to log in with the teacher ID number and student ID number, and administrators have to register and authenticate according to their ID cards. At present, the functions of the platform are divided into corpus experience area, practice area, information mutual assistance area, and acceptance area. The corpus experience area is the area where platform personnel perform functions such as denoising, labeling, indexing, term extraction, and alignment and realize the real meaning of corpus-assisted translation. The practice area focuses on practical operations and is mainly divided into the role playing of project managers, translation managers, translators, project managers, proofreaders, typesetting personnel, and corpus administrators. Platform personnel can conduct actual drills according to their own needs and improve themselves during the drills. The information mutual assistance zone is relatively simple, and platform staff can share operation and translation experience as well as learning materials such as translation tools and corpus links. The acceptance area is the area where the management personnel are located. This area collects suggestions and opinions from platform personnel. In this way, the platform is continuously optimized, and the errors are corrected to further improve the operational level of translation workshops. The plan of the translation corpus is shown in Figure 7.

The corpus has three characteristics:

1. The corpus stores the language materials that have actually appeared in the actual use of the language, so the example sentence database should not usually be regarded as a corpus.
2. Corpus is the basic resource carrying language knowledge, but it is not equal to language knowledge.
3. Real corpus needs to be processed (analyzed and processed) before it can become a useful resource.

### 3.4. Application of English Translation Based on PSO-ANNs Model

**Figure 8:**
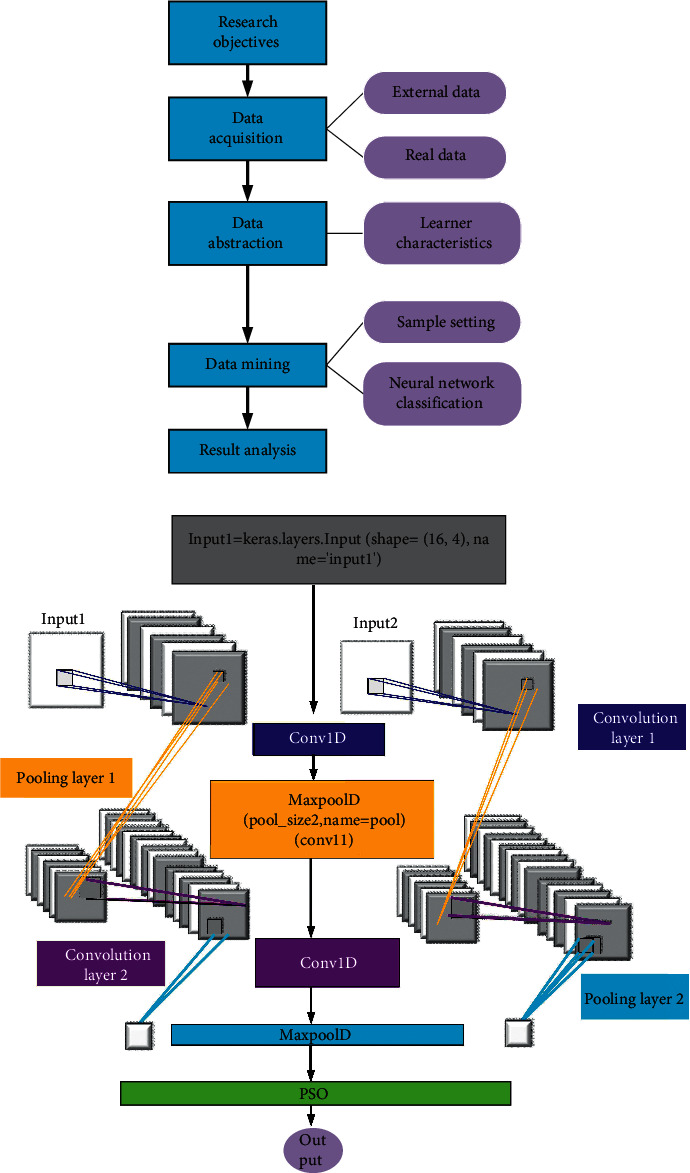
Neural network learning model. (a) Neural network. (b) Neural network with PSO.

Artificial neural network is usually composed of multiple neurons and multiple nodes according to the algorithm principles of PSO algorithm and neural network. Multilayer feedforward neural network model is the most widely used neural network model, which mainly includes input layer, output layer, and hidden layer. The input layer obtains the required information from the outside and then inputs the obtained information into the neural network for subsequent processing. The hidden layer implements processing, and the output layer can output the processed results to the desired location. The neural network learning ability analysis model shown in Figure 8 is established.

In this study, the PSO algorithm is firstly improved. The random distribution method is used to obtain inertia weights to maintain the diversity of the population and improve the search ability. At the same time, the asynchronous change strategy is used to change the value of the learning factor to strengthen the learning ability of the particles and accelerate the convergence to global optimal solution. Secondly, the improved PSO is combined with feedforward neural network algorithm. Finally, for the complex and multidimensional factors, a variety of dimensionality reduction processing methods are used to obtain the factors of the main relevant information.

Then, the following equation is used to determine the number of hidden layer nodes of the neural network model:

(4) $$\begin{array}{c} J=\sqrt{MN}. \end{array}$$

In the above equation, *J* is the number of hidden layer nodes, *M* is the number of output layer nodes, and *N* is the number of input layer nodes. According to the above equation, the relationship between the number of network training times and the number of hidden layer nodes is obtained. The optimization principle of PSO algorithm is introduced into the neural network, which enhances the global optimization capability of the algorithm. The composite algorithm uses the movement and update of particles to find the optimal solution of the neural network at the initial stage. The algorithm flow is shown below.

### 3.5. First Step

(i) Data preprocessing is implemented via the normalization method, and the purpose is reducing the noise of the original data.
  (5) $$\begin{array}{c} x_{i}=\lambda_{1}+\left(\lambda_{2}-\lambda_{1}\right)\left(\frac{z_{i}-z_{i}^{\min}}{z_{i}^{\max}-z_{i}^{\min}}\right). \end{array}$$
(ii) The output value is denormalized.

(6) $$\begin{array}{c} z_{i}=\left(\frac{x_{i}-\lambda_{1}}{\lambda_{2}-\lambda_{1}}\right)\left(z_{i}^{\max}-z_{i}^{\min}\right)+z_{i}^{\min}. \end{array}$$

*x*_*i*_ is the normalized value, *z*_*i*_ is the denormalized value, *λ*_1_ is the lower limit, *λ*_2_ is the upper limit, *z*_*i*_^max^ is the maximum value in the original data, and *z*_*i*_^min^ is the minimum value in the original data.

The second step is setting the neural network related parameters.

- Determine the number of hidden nodes.
- Determine the hidden layers.
- Determine the activation function by selecting the Sigmoid function as the activation function.

(7) $$\begin{array}{c} \delta \left(t\right)=\frac{1}{1+e^{-t}}. \end{array}$$

The third step is PSO initial position and speed setting.

The initial position is

(8) $$\begin{array}{c} X_{i}=\left(x_{i1},x_{i2},\ldots ,x_{in}\right). \end{array}$$

The initial speed is

(9) $$\begin{array}{c} V_{i}=\left(v_{1},v_{i2},\ldots ,v_{i\;d}\right). \end{array}$$

Optimal location record is

(10) $$\begin{array}{c} P\text{g}=\text{min}\left\{P_{0},P_{1},\ldots ,P_{s}\right\}. \end{array}$$

The fourth step is the network output:

(i) Hidden output layer
  (11) $$\begin{array}{c} t_{h}=\sum_{i}\left(a_{ih}\right)x_{i}-\theta_{h},\\ H_{h}=f\left(t_{h}\right)=\frac{1}{1+e^{-t_{h}}}. \end{array}$$
(*t*_*h*_) is the weighted sum of the input of the *h* layer. *a*_*ih*_ is the connection parameter between the *i* layer and the *h* layer. *x*_*i*_ is the activation value of the *i* layer. *θ*_*h*_ is the offset of the *h* layer.
(ii) Output layer is
  (12) $$\begin{array}{c} t_{j}=\sum_{h}\left(a_{hj}\right)H_{h}-\theta_{j},\\ Y_{j}=f\left(t_{j}\right)=\frac{1}{1+{\text{e}}^{-t_{j}}}. \end{array}$$
(*t*_*j*_) is the weighted sum of the input of the *j*-th layer. *Y*_*j*_ is the output of the *j*-th layer.
(iii) Calculate reverse difference:
  (13) $$\begin{array}{c} \delta_{j}=Y_{j}\left(1-Y_{j}\right)\left(T_{j}-Y_{j}\right),\\ \delta_{h}=H_{h}\left(1-H_{h}\right)\sum_{j}w_{hj}\delta_{j}. \end{array}$$
(iv) The weight matrix Δ*w* and bias vector Δ*θ* are calculated and updated:

(14) $$\begin{array}{c} \Delta w_{hj}=-\eta \delta_{j}H_{h},\\ \Delta \theta_{j}=-\eta \delta_{j},\\ w_{hj}=w_{hj}^{\prime }+\Delta w_{hj},\\ \theta_{j}=\theta_{j}^{\prime }+\Delta \theta_{j}. \end{array}$$

In the above equation, **w**_*hj*_ and *θ*_*j*_ represent the new weight matrix and bias vector, respectively. **w**_*hj*_′ and *θ*_*j*_′ are the original weight matrix and bias vector, respectively.

The fifth step is as follows:

- Calculate particle fitness.
- Update particle position and velocity.
- PSO-ANNs model.

The mobile edge computing system designed in this study is divided into three parts. The first part is the mobile device, the second part is the edge server, and the third part is the cloud server. In this work, the mobile client is used to collect data through mobile phone software and upload it to the edge server. The edge server extracts the discriminant information through the projection matrix trained by the cloud server and compares it with the relevant information to obtain the relevant results and returns it to the mobile terminal. Its related feature information will be sent to the cloud server as a new training sample. The cloud server uses the projection matrix and feature information in the training database to send it to the edge server. In this study, Alibaba Cloud is selected as the cloud server, the hierarchical discriminant analysis algorithm proposed in this study is adopted to train the projection matrix obtained in the database, and the projection matrix was applied to extract the feature information of the data in the database and send it to the edge server for identification. A large amount of identifiable data is stored in the cloud server and trained using a hierarchical discriminant analysis feature extraction algorithm, and the projection matrix and feature information of all images are obtained, which is sent to the edge server as the basis for translation recognition.

### 3.6. Model Parameter Settings and Dataset Construction

1. Model parameter setting is to set the two input objects. Input1 is undertaken as an example. Input2 and input1 perform the same operation, and the input form of the defined data is input1 = keras.layers.Input(shape=(16,4),name = 'input1′), which represents a 16  ×  4 matrix. Then the data goes into the convolutional layer, where input1 is renamed, input1 is named Convolution1, and input2 is named Convolution2. After the operation of the convolution layer, the data will be subjected to a secondary operation in the pooling layer. After the operation of the pooling layer is completed, the data processing of the CNN part is basically completed, and then the data processed by the CNN is used as the input of the particle swarm algorithm. The ReLU function is used as the activation function.
2. Construction of the dataset: It can be seen from the previous data acquisition and preprocessing that, to fully train the model to achieve the true prediction of the problem, the data needs to be divided into training set and test set. The training set is used for model training and model parameter determination, and the test set is used to finally test the performance of the model. In this work, the training set and test set were divided according to the ratio of 9 : 1.

### 3.7. Model Training Environment Configuration

This research experiment is completed under the Ubuntu 16.04 operating system. The model programming language uses Python 3.6, and the compilation environment uses software Anaconda. The training in this study is implemented based on the Keras framework. Keras is a Python-based deep learning framework built on TensorFlow 2.0, which can easily define and train almost all types of deep learning models.

## 4. Results and Analysis

### 4.1. Experiment Simulation Results of Edge Collaboration

**Figure 9:**
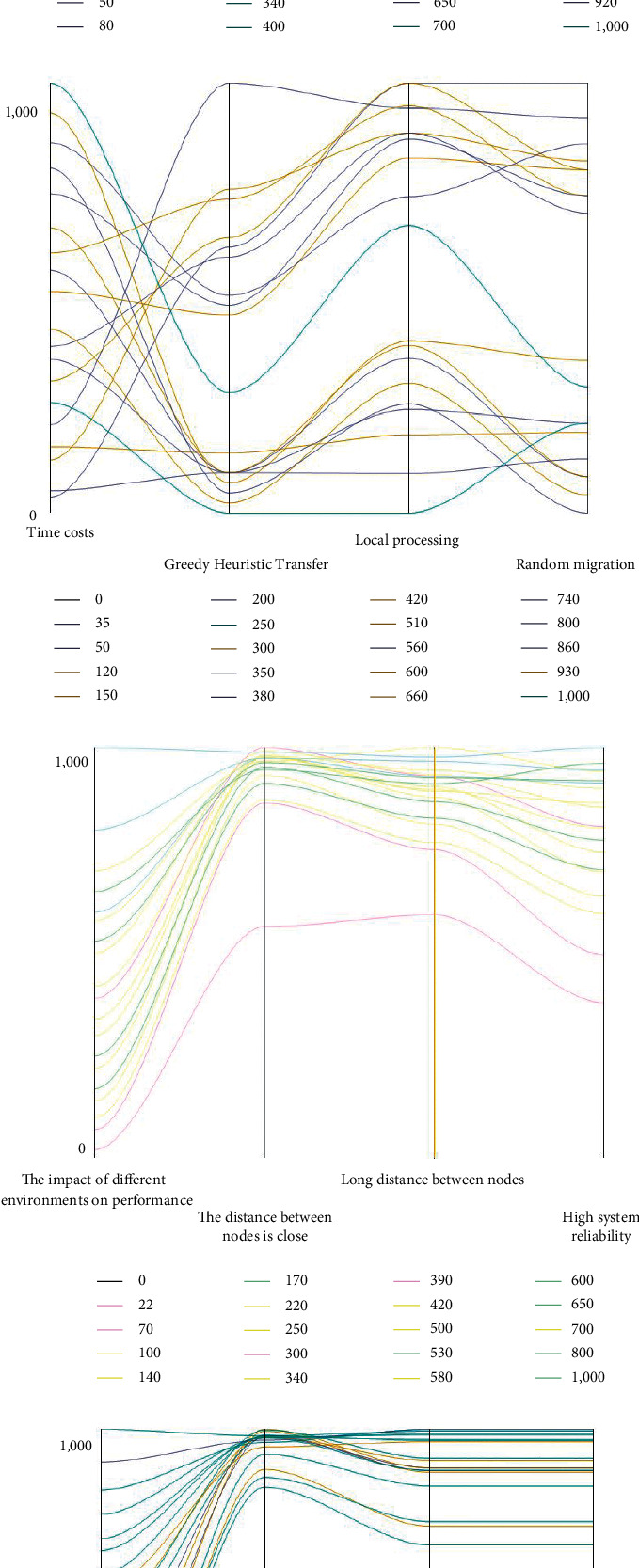
The experiment simulation results of the edge collaboration. (a) Comparison of system throughput; (b) comparison of time and cost of completing tasks; (c) impact of deployment environment on system performance; (d) impact of different thresholds on system throughput.

The experiment simulation results of the edge collaboration are illustrated in Figure 9. The curves in different colors represent different simulated values.

**Figure 10:**
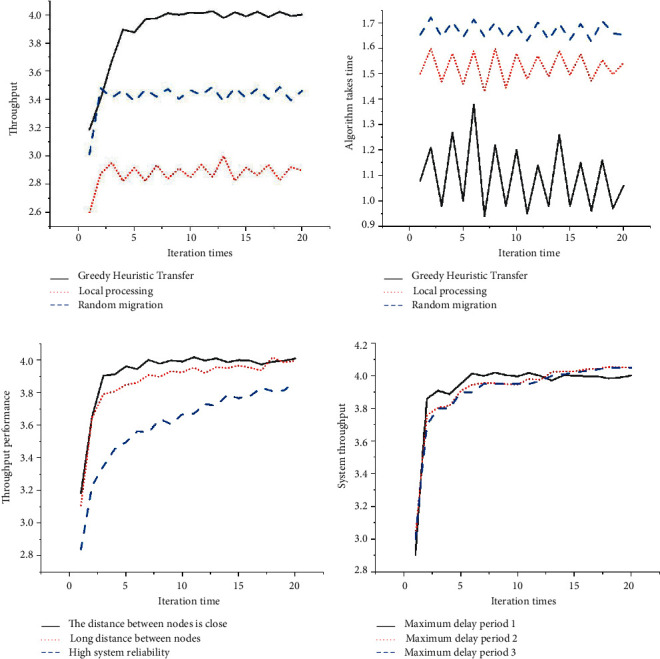
The experiment simulation results of the edge collaboration. (a) Comparison of system throughput; (b) comparison of time and cost of completing tasks; (c) impact of deployment environment on system performance; (d) impact of different thresholds on system throughput.

Further simplification of the data in Figure 9 during the course of the study resulted in the data plots in Figures 10(a)–10(d), respectively.

In Figures 9 and 10, A represents the throughput comparison of RM, CLM, and GHM algorithms, while B represents the task completion time and cost of the three algorithms. C represents the influence of environmental factors on the new performance of the system, and D represents the influence of different thresholds set by the three algorithms on throughput.

Figures 9(a) and 10(a) illustrate the comparison of the throughput of three different migration algorithms. There are three different migration algorithms, RM, CLM, and GHM, when the system runs stably. The system throughput of the third algorithm has increased by 12% and 50%, respectively, in contrast to that of the other two algorithms. Therefore, the GHM algorithm shows better performance than the other two task migration methods regarding the system stability.

Figures 9(b) and 10(b) show the comparison of the time cost of the three algorithms. The average time for the CLM algorithm requires the longest time to complete the task, for it is affected by the computing power of the terminal. The comparison shows that the average time for the GHM algorithm to complete the task is reduced by 60% compared to the CLM algorithm. Therefore, the GHM algorithm shows the best stability among the three different algorithms.

Figures 9(c) and 10(c) show the comparison results on the impacts of different deployment environments on system performance. The relatively obvious impact on system throughput performance is during task migration, and the deployment distance among nodes has a small impact on system throughput performance. Therefore, the possibility of task transmission among nodes should be prioritized. In the three different deployment environments, the system stability is 1.4 × 10^8^, 2.8 × 10^8^, and 6.5 × 10^8^, respectively. The data shows that the third algorithm is the most degraded, followed by the first two. Figures 9(d) and 10(d) show the comparison results on the impacts of different maximum delay time on system throughput. The system stability has a small fluctuation when the values are 1, 2, and 5 when other parameters remain unchanged. In other words, the difference has minimal impact on the performance of task migration algorithm. (B) shows simulation results of MEC system migration.

**Figure 11:**
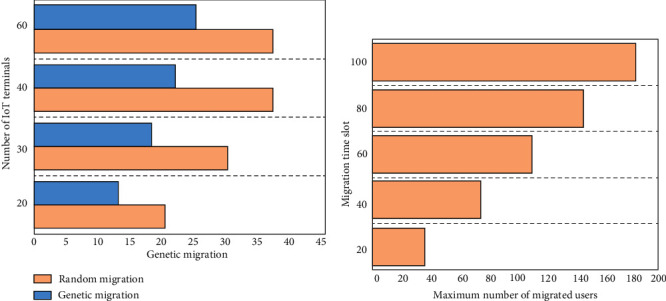
Time slot and maximum number of mobile terminals. (a) Maximum number of mobile terminals when time slot is fixed; (b) mobile time slot length and maximum number of mobile terminals.

The simulation results on the relationship between the total number of migrated users and the migration rate are shown in Figure 11.

In Figure 11, A represents the comparison of the maximum number of mobile terminals of traditional migration and genetic migration in fixed time slot, and B represents the length of mobile time slot and the maximum number of mobile terminals.

Figure 11(a) shows the number of IoT terminals migrated by CLM, GHM, and RM algorithms when the system time slot length is fixed to 20 ms with different total users. GHM algorithm shows the user migration rate of 100% when the number of terminal users is 20 or 30. There is no user migration under the CLM algorithm (the migration rate is 0), and the user migration rate under the RM algorithm is 66% and 63%, respectively. The user migration rate under the GHM algorithm is 93% and 62%, respectively, after the number of terminal users rises to 40 and 60. That under the CLM algorithm is still 0, and that under the RM algorithm is 56% and 37%, respectively. Therefore, the migration rate of RM algorithm is lower than that of GHM algorithm as the number of users increases.  Figure 11(b) shows the results of the maximum number of users migrated in different system time slot lengths. The migration time slot increases from 20 ms to 100 ms, and the maximum numbers of users included in the GHM algorithm are 21, 38, 54, 74, and 92, respectively.

**Figure 12:**
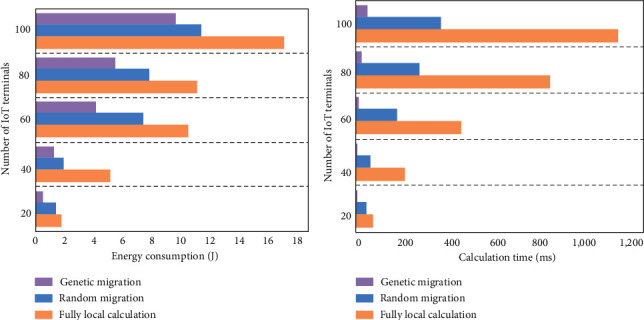
The comparison on performances of different migration algorithms. (a) Comparison on energy consumption; (b) comparison on computing time.

The comparison on performances of different migration algorithms is shown in Figure 12.

Figure 12(a) represents the comparison of energy consumption of the three migration algorithms, and 12(b) represents the comparison of computing time of the three migration algorithms.

Figure 12(a) shows the energy consumption comparison of three algorithms. The system time slot length is also set to 20 ms, and the total number of users is set to increase at 10–80 gradient. The genetic algorithm saves about 20% of energy compared to the RM algorithm when the number of system users is less than 40, and its energy-saving ratio is about 59%. However, the energy-saving ratio of the genetic algorithm begins to decrease when the number of users in the system is greater than 40. The energy-saving percentage dropped again when the total number of system users increases to 50. The trend shows that the energy-saving ratio of the genetic algorithm is lower than that of the CLM algorithm and the RM algorithm, so the genetic migration algorithm shows high energy-saving efficiency.

Figure 12(b) shows the comparison of the time cost simulation experiment results of the three algorithms. The time required for the hybrid genetic migration algorithm is much lower than the time required for CLM algorithm when the number of system users does not exceed 40. The time required for execution of the migration algorithm increases fast when the number of system users is greater than 40. Therefore, the genetic algorithm proposed in this study is more efficient than the RM algorithm and the CLM algorithm.

### 4.2. PSO-ANNs Simulation Results

**Figure 13:**
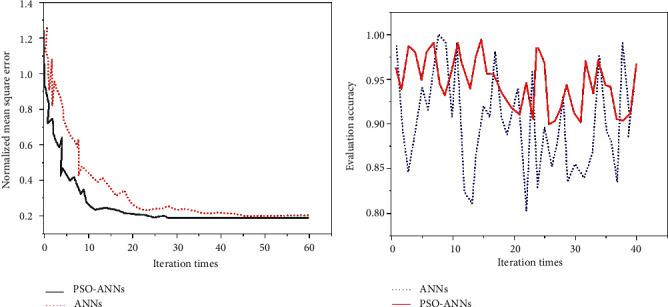
English translation teaching quality evaluation results. (a) Mean square error comparison; (b) simulation accuracy comparison.

The PSO-ANNs model is used to verify the teaching effect of English translation. First, the sample collection of students' English translation learning characteristics is completed, and then it is proceeded in two steps. The first step uses PSO algorithm to train the neural network. The second step is evaluating and testing the validity of the model. The algorithm dataset is from Tianchi Datasets of Alibaba, and 2,000 students' English translation data are taken as the dataset of the algorithm in this research. However, due to space issues, the datasets are not provided in this article. The established model evaluates the quality of English translation teaching and compares it with ANNs model. The results are shown in Figure 13.

**Figure 14:**
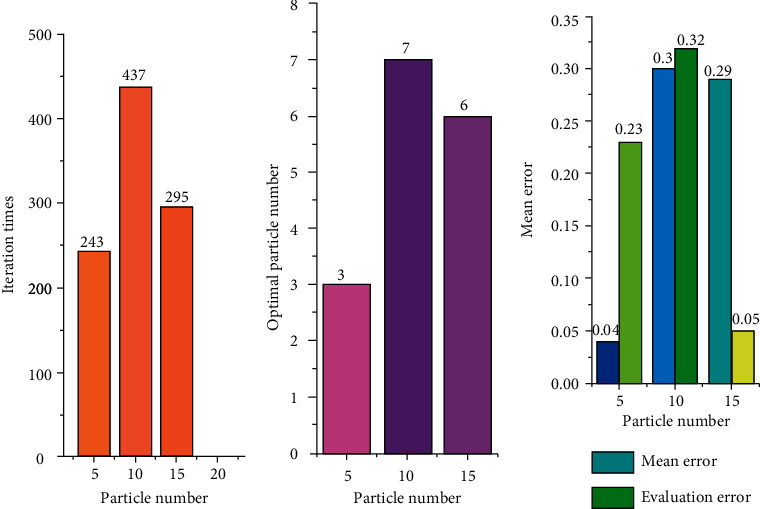
Simulation results of English translation teaching effect. (a) Number of iterations; (b) optimal particle number; (c) error.

Figure 13(a) shows that ANNs reach convergence after about 45 iterations, while the PSO-ANNs model only needs about 30 iterations to reach convergence. The addition of PSO algorithm can effectively improve the convergence speed of ANNs neural network and reduce model training time.  Figure 13(b) shows that the evaluation accuracy of the PSO-ANNs model with the PSO algorithm is due to the ANNs, indicating that the PSO algorithm can improve the accuracy of the ANNs network. Then, the PSO-ANNs model is used to verify the effect of English translation teaching, and the result is shown in Figure 14.

The training results of the algorithm are shown in Figure 14. Under the same experimental conditions, the model error is 0.23 when the number of particles is 5, and the model error is 0.32 when the number of particles is 10. When the number of particles is 15, the error of the model is 0.05, and the performance of the model is the best at this time. It means that, within a certain range, the more the number of particles, the better the algorithm performance.

## 5. Conclusion

In the context of the IoT based on edge computing, this study analyzes the English translation teaching practice, corpus construction methods, and task transfer algorithm technology. Combined with the system task transfer algorithm of the MEC scenario, an English-Chinese/Chinese-English bilingual translation corpus was constructed, and a two-way interactive online English translation course is designed. GHM algorithm shows better performance than CLM algorithm and RM algorithm in actual English teaching. In addition, the addition of PSO algorithm can effectively improve the convergence speed of the neural network, reduce the model training time, and improve the accuracy of the neural network. The neural network is trained by the PSO algorithm, and the optimal solutions of different particle populations are obtained. Under the same experimental conditions, the model error is 0.23 when the number of particles is 5; the model error is 0.32 when the number of particles is 10; and the error of the model is 0.05 when the number of particles is 15. The model performs best at this point. However, this study also has some shortcomings. Firstly, it only focuses on two-way interactive online English translation courses and does not discuss preclass preview and after-class maintenance. Secondly, due to the limited resources and less test data of the system, further exploration is needed in the future to supplement and improve the application of the proposed task transfer algorithm in English practice classroom research.

In future research, it will discuss the application of edge computing technology in “interactive” translation teaching mode. It is believed that the combination of edge computing technology and “interactive” translation teaching mode can make up for some deficiencies in traditional translation teaching, which contributes to the realization of translation teaching goals.

## Figures and Tables

**Table 1:** Experiment parameter setting of edge collaboration.

Parameter	*K*	Number of base stations	*P*_*ij*_	*c*_*1*_	*c*_*2*_	*S*_*ni*_	*a*_*ni*_
Value	1,000	5	0–0.2	0.1	0.3	100–1,000	100–900

**Table 2:** The simulation experiment parameters for MEC calculation migration.

Parameter	*K*	*T* (ms)	*C*_*k*_	*σ*	*B*	*P*_*k*_	*S*_*k*_	*e*_*k*_
Value	20, 40, 60, 80	20	500–1,000 period/bit	10^−9^ W	10 MHZ	100–500 mW	1–10 GHZ	0–2 × 10^−10^

## Data Availability

The data used to support the findings of this study are included within the article.

## References

[1] Xu Z., Shi Y. (2018). Application of constructivist theory in flipped classroom - take college English teaching as a case study. *Theory and Practice in Language Studies*.

[2] Du Y. (2018). Discussion on flipped classroom teaching mode in college English teaching. *English Language Teaching*.

[3] Thomson D., Brooks S., Nuspl M., Hartling L. (2019). Programme theory development and formative evaluation of a provincial knowledge translation unit. *Health Research Policy and Systems*.

[4] Gafurovna R. Z. (2021). Translation theory: object of research and methods of analysis. *International Journal of Thermal Sciences*.

[5] Wenjin L. (2020). Research on the application of artificial intelligence in German translation software design from the perspective of functional translation theory. *Solid State Technology*.

[6] Kingsnorth S., Orava T., Parker K., Milo-Manson G. (2020). From knowledge translation theory to practice: developing an evidence to care hub in a pediatric rehabilitation setting. *Disability & Rehabilitation*.

[7] Chen J. (2019). Strategies for improving the effectiveness of English translation teaching in higher vocational colleges. *Based on Data Mining*.

[8] Wongranu P. (2017). Errors in translation made by English major students: a study on types and causes. *Kasetsart Journal of Social Sciences*.

[9] Abbas N., Zhang Y., Taherkordi A., Skeie T. (2017). Mobile edge computing: a survey. *IEEE Internet of Things Journal*.

[10] Zhang F. (2017). Quality-improving strategies of college English teaching based on microlesson and flipped classroom. *English Language Teaching*.

[11] Dubskikh A., Butova A. (2019). *Virtual educational environment as one of the perspective technologies of e-learning in foreign language teaching*.

[12] Wu A., Song E. (2020). Distribution of teaching surveillance video via edge computing. *Internet Technology Letters*.

[13] Sun J. (2020). Research on resource allocation of vocal music teaching system based on mobile edge computing. *Computer Communications*.

[14] Bao L., Yu P. (2021). Evaluation method of online and offline hybrid teaching quality of physical education based on mobile edge computing. *Mobile Networks and Applications*.

[15] Sun Y., Wang J. (2020). English translation of neural network algorithm based on particle swarm optimization. *Science Technology and Engineering*.

[16] Olguín J., Pietraszko J. M., Jorge R. R. (2018). Particle swarm optimization as a. *New Measure of Machine Translation Efficiency*.

[17] Malyuga E. N., Krouglov A., Tomalin B. (2018). Linguo-cultural competence as a cornerstone of translators’ performance in the domain of intercultural business communication. *XLinguae*.

[18] Mellinger C. D. (2017). Translators and machine translation: knowledge and skills gaps in translator pedagogy. *The Interpreter and Translator Trainer*.

[19] De Sutter G., Lefer M.-A. (2020). On the need for a new research agenda for corpus-based translation studies: a multi-methodological, multifactorial and interdisciplinary approach. *Perspectives*.

[20] De Bastos M. D. C. H. (2019). Procuração/power of attorney: a corpus-based translation-oriented analysis. *Translation Spaces*.

[21] Farahani M. (2021). Corpus Linguistics for translation and contrastive studies: a guide for research. *Interpreting*.

[22] Marjanovic M., Antonic A., Zarko I. P. (2018). Edge computing architecture for mobile crowdsensing. *IEEE Access*.

[23] Xu Y., Nascimento N. M. M., de Sousa P. H. F. (2021). Multi-sensor edge computing architecture for identification of failures short-circuits in wind turbine generators. *Applied Soft Computing*.

[24] Rasheed A., Chong P. H. J., Ho I. W.-H., Li X. J., Liu W. (2019). An overview of mobile edge computing: architecture, technology and direction. *KSII Trans. Internet Inf. Syst.*.

[25] Ren J., Pan Y., Goscinski A., Beyah R. A. (2018). Edge computing for the internet of things. *IEEE Network*.

[26] Sittón-Candanedo I., Alonso R. S., Corchado J. M., Rodríguez-González S., Casado-Vara R. (2019). A review of edge computing reference architectures and a new global edge proposal. *Future Generation Computer Systems*.

[27] Yuan Q., Zhou H., Li J., Liu Z., Yang F., Shen X. S. (2018). Toward efficient content delivery for automated driving services: an edge computing solution. *IEEE Network*.

[28] Mach P., Becvar Z. (2017). Mobile edge computing: a survey on architecture and computation offloading. *IEEE Communications Surveys & Tutorials*.

[29] Wang T., Zhang G., Liu A., Bhuiyan M. Z. A., Jin Q. (2018). A secure IoT service architecture with an efficient balance dynamics based on cloud and edge computing. *IEEE Internet of Things Journal*.

[30] Shi Y., Qu H., Zhao J. (2017). Dual connectivity enabled user association approach for max-throughput in the downlink heterogeneous network. *Wireless Personal Communications*.

[31] Bisiada M. (2018). Translation and editing: a study of editorial treatment of nominalisations in draft translations. *Perspectives*.

[32] Bisiada M. (2018). Editing nominalisations in English−German translation: when do editors intervene. *The Translator*.

[33] Ercan G., Haziyev F. (2019). Synset expansion on translation graph for automatic wordnet construction. *Information Processing & Management*.

[34] Jones H. (2019). Searching for statesmanship: a corpus-based analysis of a translated political discourse. *Polis: The Journal for Ancient Greek and Roman Political Thought*.

[35] Hoek J., Zufferey S., Evers-Vermeul J., Sanders T. J. M. (2017). Cognitive complexity and the linguistic marking of coherence relations: a parallel corpus study. *Journal of Pragmatics*.

